# Use of Traditional Chinese Medicine for Patients Diagnosed with Postpartum Depression: A Nationwide Population-Based Study

**DOI:** 10.1155/2020/7060934

**Published:** 2020-07-20

**Authors:** Jung-Miao Li, Cheng-Li Lin, Ke-Ru Liao, Chung-Chih Liao

**Affiliations:** ^1^Graduate Institute of Chinese Medicine, College of Chinese Medicine, China Medical University, Taichung 40402, Taiwan; ^2^Department of Chinese Medicine, Show Chwan Memorial Hospital, Changhua 50008, Taiwan; ^3^Management Office for Health Data, China Medical University Hospital, Taichung 40447, Taiwan; ^4^College of Medicine, China Medical University, Taichung 40402, Taiwan; ^5^Department of Neurology, Yuanlin Christian Hospital, Yuanlin 51052, Taiwan

## Abstract

Postpartum depression (PPD) is one of most common postnatal complications, affecting approximately 10%–15% of women after childbirth annually. Traditional Chinese medicine (TCM) has been gaining popularity as the choice of treatment for PPD in Taiwan. Hence, our aim was to analyze the utilization of TCM among PPD patients in Taiwan. A cross-sectional study was conducted using a random sample of one million beneficiaries selected from the Taiwanese National Health Insurance Research Database. We identified patients with PPD who had received either TCM treatment or non-TCM treatment from the database during 2000–2012. Multivariate logistic regression analysis was used to identify the factors associated with the use of TCM. A total of 653 patients with PPD were enrolled. The majority of patients with PPD were 26–30 years old, lived in a highly urbanized area of Taipei, had a monthly income <20,000 NT$, and were private enterprise employees. Around 52.7% of PPD patients had the motivation to seek TCM services. Younger women, who resided in central and southern Taiwan and who had used TCM one year before PPD diagnosis, were more likely to use TCM services. PPD patients who underwent TCM treatment had a lower overall medical expenditure than non-TCM users. Most TCM users chose simple Chinese herbal medicine. The coexisting factors that made PPD patients to seek TCM services were respiratory or oral infections. We demonstrated the characteristics of those that seek TCM for PPD, which may provide useful insights to health care providers towards resource allocation.

## 1. Introduction

Childbirth is usually a unique and unforgettable experience for most women of childbearing age. However, some factors interfere with the positive aspects of mother-child interactions of new mothers, including the exhausting process of childbirth, postpartum physical uncomfortable conditions, breastfeeding stress, lifestyle changes, and relationship dynamic changes [1, 2]. Hence, some women suffer from fluctuating moods postpartum. Postpartum depression (PPD) is a depressive disorder often occurring within one year of delivery [3]. It is one of the most common postnatal complications, during which the person with PPD can experience sadness, worthlessness, or hopelessness, which could potentially result in maternal and familial negative consequences, disabilities, or life-threatening situations [4, 5]. PPD affects about 10%–15% of new, adult mothers annually, which often leads to a substantial humanistic burden on affected mothers, their partners, and their other children [6, 7].

Psychotherapy and antidepressant medications are the gold-standard treatment for PPD [8]. Although antidepressant medications are convenient treatments for PPD, their potentially troubling side effects can affect normal routine activities. These side effects include weight gain, sleep disturbances, and sexual dysfunction, which should be considered before taking long-term antidepressants [9]. Furthermore, some reports revealed that different levels of antidepressant drugs may transfer into breast milk, which leads to the mother having to make the choice whether she should receive medication therapy at the risk of her infant's health [10, 11]. Hence, some patients consider other safe, effective, economic therapies to replace conventional western medicine (WM).

Traditional Chinese medicine (TCM), which includes Chinese herbal medicine (CHM), acupuncture, and manipulative therapies, has been used for depressive disorders for more than 5000 years [12] and now has extensive scientific evidence supporting its efficacy. A systematic review and meta-analysis report included 443 PPD patients who received acupuncture and 444 PPD patients who did not, with the results showing that acupuncture led to improvements on the Hamilton Depression Scale and had an effective rate [13]. Yang et al. concluded that CHM could significantly reduce PPD symptoms compared to antidepressants [14]. However, there is still little evidence about the factors involved in TCM use by the PPD population based on a large sample size.

Since 1995, the National Health Insurance (NHI) program has offered insurance coverage for TCM and WM for nearly 99.6% of the 23 million residents of Taiwan [15]. The Bureau of the NHI has cooperative agreements with nearly 92% of all clinics and 97% of all hospitals [16]. Intact clinical data of TCM use by women with PPD were collected from the Taiwanese National Health Insurance Research Database (NHIRD), which is considered to reflect real-world evidence of TCM clinical practice for research purposes [17]. Thus, this study aimed to apply the NHIRD information to explore the determinants of TCM use in patients with PPD.

## 2. Materials and Methods

### 2.1. Data Source

We conducted a nationwide, population-based, cross-sectional analysis by using the Longitudinal Health Insurance Database 2000 (LHID2000), which comprises a random sample of one million subjects from the NHIRD longitudinally linked data available from 1997 through 2013. This database contains sociodemographic data, dates of visits, International Classification of Diseases, Ninth Revision, Clinical Modification (ICD-9-CM) diagnostic codes, complete prescription details, and expenditure incurred by the beneficiaries. Data of detailed diagnoses and treatments provided by physicians were included. These files were linked by using a scrambled, anonymous identification number for each subject to obtain the longitudinal medical history. This study was approved by the Institutional Review Board of China Medical University in central Taiwan (CMUH109-REC2-031).

### 2.2. Study Population

**Figure 1:**
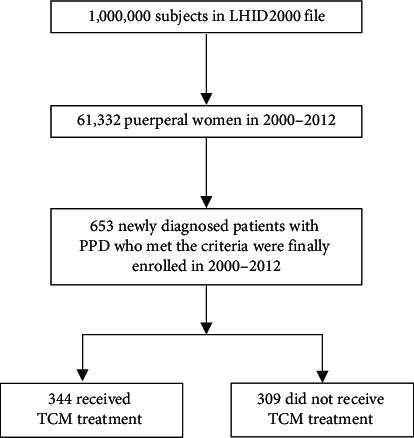
Flowchart for study subject enrolment. LHID2000: Longitudinal Health Insurance Database 2000. TCM: traditional Chinese medicine.

We selected participants covering the period from January 1, 2000, to December 31, 2012, by using the LHID2000. The flow chart is shown in Figure 1. First, we identified the postnatal women included in the LHID2000 (*n* = 61,332). Any diagnosis of depression, in which at least two ambulatory or inpatient claims were made by psychiatrists who prescribed antidepressants for treatment within one year after delivery was defined as PPD (ICD-9-CM code: 296.2, 296.3, 296.82, 300.4, 309.0, 309.1, 311). Among these, we excluded subjects that fell under the criteria of being younger than 20 years of age, who had a previous history of depression, or had incomplete medical records. Finally, a total of 653 women newly diagnosed with PPD that met our criteria were enrolled. Subjects who had visited a Chinese medicine clinic and received TCM treatment at least once after being newly diagnosed with PPD were deemed “TCM users,” and the rest were deemed “non-TCM users.” The date of first TCM usage after the diagnosis date of PPD was defined as the index date for the TCM cohort. The follow-up period of all participants was within one year from the date of initial diagnosis.

### 2.3. Sociodemographic Characteristics

The sociodemographic factors included age, insured amount, urbanization level, residential area, and insured unit categories. Adult patients were further divided into six subgroups: 20–25, 25–30, 30–35, 35–40, 40–45, and >45 years. The amount of insurance premium, determined from the individual working salary, was classified into four levels: < 20,000, 20,000–39,999, 40,000–59,999, and >60,000 NT$/month. Furthermore, the urbanization level of the townships in Taiwan was categorized according to educational level of the population, population density, ratio of elderly people, and occupation in general. The residential areas of the study population comprised of 6 areas: northern area, Taipei, central area, southern area, eastern area, and Kao-Ping area. The insured units included government, school, private enterprise, occupational members, farmers and fishermen, low-income household, and veterans. Furthermore, the season during which the delivery took place was included as a factor. We used the official definition of seasons of the Taiwanese Central Weather Bureau (CWB) as spring (March–May), summer (June–August), autumn (September–November), and winter (December–February).

### 2.4. Medical Factors

TCM use one year prior to PPD diagnosis was considered. Baseline comorbidities with at least two ambulatory or inpatient claims were also considered in order to qualify for the present study, including diabetes mellitus (ICD-9-CM 250), hypertension (ICD-9-CM 401–405), hyperlipidemia (ICD-9-CM 272), stroke (ICD-9-CM 430–438), coronary artery disease (ICD-9-CM codes 410–414), cirrhosis (ICD-9-CM 571), and renal disease (ICD-9-CM 585).

### 2.5. Medical Expenditure

We analyzed the total number of medical expenditures within one year after the first diagnosis date of PPD, which took TCM and WM ambulatory care into consideration. Total medical expenditure included consultation fees, charges for treatment and medical supplies, diagnosis fees, and drug fees.

### 2.6. Coexisting Diseases of Outpatient Department Visits after PPD Diagnosis

We analyzed the frequency distribution of coexisting diseases of OPD (outpatient department) visits after PPD diagnosis in the TCM cohort and non-TCM cohort based on the ICD-9 codes.

### 2.7. Statistical Analysis

The continuous variables were evaluated using means and standard deviations (SD), whereas categorical variables were evaluated using numbers and percentages. To compare the differences in continuous variables between TCM users and non-TCM users, Student's *t* test was used, whereas the chi-squared test was used to analyze the categorical variables. Furthermore, the adjusted odds ratio (OR) and 95% confidence interval (CI), calculated using a multivariate logistic regression analysis, were used to explore the determinants of TCM use. A *p* value <0.05 was considered statistically significant. All statistical analyses and figures were performed using SAS software, version 9.4 (SAS Institute Inc., Cary, NC, U.S.A.).

## 3. Results

### 3.1. Factors Associated with TCM Use in Patients with PPD

We identified a total of 653 patients with newly diagnosed PPD who met the study criteria between 2000 and 2012 in the LHID2000 (Figure 1). Among the included subjects, 344 patients (52.7%) received TCM treatment and 309 patients (47.3%) did not receive TCM treatment.

Characteristics of the TCM user and non-TCM user cohorts of patients with PPD are presented in Table 1. The mean duration between the delivery dates and initial diagnosis of PPD was approximately 0.44 years. The mean age of PPD patients was 31.2 years. The highest proportion of PPD patients in both cohorts were in the 26-30-year age group, followed by the 31–35-year-old and 20–25-year-old groups. There were no substantial differences in the insured amounts, urbanization levels, or insured units between the two groups. The difference between the number of patients with PPD was not significantly different among spring, summer, autumn, and winter. The most common baseline comorbidity was cirrhosis, followed by hypertension and coronary artery disease. The prevalence of all baseline comorbidities was similar in both groups. Furthermore, using a multivariate-adjusted analysis, we observed that patients with PPD who were in the 20-35-year age group, TCM users one year prior to the PPD diagnosis, who resided in the central and southern area, were more likely to use TCM services.

### 3.2. TCM Visits and Medical Expenditure

The mean duration between the initial diagnosis of PPD and the first TCM treatment was approximately 0.31 years. The mean number of OPD visits to a TCM clinic within one year of PPD diagnosis was 5.58 (Table 1).

The total medical expenditure of the TCM users and non-TCM users with PPD within a one-year follow-up after the initial diagnosis of PPD is compared in Table 1. The total (average) cost in TCM users was lower than that of non-TCM users; nevertheless, the difference in average cost between these two groups was minimal (43,071 NT$ vs. 47,395 NT$, *p*=0.74).

### 3.3. Types of TCM Use

We categorized the most frequent types of TCM use in Taiwanese patients into 3 categories: simple CHM treatment, simple acupuncture/manipulative treatment, and combination of CHM/acupuncture/manipulative treatment. As shown in Table 2, in the TCM user cohort, 71.5% of patients with PPD were treated with CHM treatment only, 2.3% were treated with acupuncture or manipulative treatment only, and 26.2% were treated with combination treatment within a one-year follow-up.

### 3.4. Distribution of Coexisting Diseases in TCM and Non-TCM Visits in Patients with PPD

We observed which infectious diseases commonly coexisted as the reason for OPD visits after the patients' diagnosis of PPD, irrespective of TCM use by the patients (Table 3). The top 5 coexisting diseases of PPD patients who used TCM were respiratory or oral infection-related conditions, including acute upper respiratory infections (5.83%), acute nasopharyngitis (3.79%), acute tonsillitis (3.5%), dental caries (3.21%), and acute pharyngitis (2.04%).

## 4. Discussion

The determinants for TCM use for PPD patients in a nationwide population have scarcely been investigated in the past. The NHIRD offered a sufficient population sample size and a great deal of information, eliminating the bias associated with a limited sample size. This means that the data can be considered close to real-world evidence and can be used as an appropriate source to assess the disease and treatment efficacy. In addition, it is worth noting that the TCM practice is only performed by well-trained, qualified TCM doctors in Taiwan, which guarantee the most suitable treatment for patients, as well as accurate and valuable research results.

We reported that the incidence of PPD was approximately 1% retrieved from the NHIRD, which was lower compared with the previous studies [18, 19]. According to a previous study involving NHI data, around 25.5% and 26.8% of people sought out TCM treatment at least once in South Korea and Taiwan, respectively [20]. In the TCM user population, women have a higher proportion of use (adjusted OR 1.47–1.62) than men [21]. This could potentially be attributed to the consideration of the adverse effects of current WM therapy for PPD and the worry about the potential negative impact of chemicals affecting their baby through breastfeeding, leading some women to seek alternative therapy or natural therapy for treatment. The present findings revealed that more than fifty percent of patients with PPD had the desire to consult TCM services to help treat the disease in addition to WM. The proportion of TCM use for this disease is higher than the average use of TCM, which is why TCM use in PPD is noteworthy for TCM physicians and affects health resource allocation. In addition, the prevalence of TCM outpatient visits may be underestimated, because some patients visiting TCM doctors preferred to look for decoction formulations of Chinese medicine, which were self-paid and not covered by the NHI program (in Taiwan, the NHI program only supplies medicine in scientifically concentrated powder or granule form, which is more convenient than traditional decoction formulations of CHM).

Our findings revealed the largest proportion of TCM use was simple CHM therapy, followed by combination therapy, including CHM, acupuncture, and manipulative therapies. In the Chinese society, postpartum women undergo postpartum conditioning (also called “doing the month”) with CHM therapy and follow the traditional Chinese diet and culture [22, 23]. For example, *Sheng-Hua-Tang* (a well-known Chinese formula) has been widely applied to resolve blood stasis, promote blood flow, and eliminate lochia, when used within the first week of the postpartum period). *Si-Wu-Tang*, also a widely known Chinese formula, is widely used to increase blood supply and regulate menstruation after delivery. Both have been proven to improve women's physical and mental health conditions after child birth [22, 24, 25].

Previous studies also showed that acupressure, acupuncture, and moxibustion therapy can be used to improve postpartum mental health, including anxiety, depression, and sleep quality [26–28]. Nevertheless, our present findings showed that only 2.3% patients with PPD received simple acupuncture therapy. This phenomenon merits further exploration.

Empirical TCM theories emphasized the relationship and interactions between the human body and the environment [29]. Interestingly, climate variations might lead to the incidence of different disease tendencies and different behaviors of TCM usage [30, 31]. Hence, we were curious about the association between seasonal variations of delivery times of PPD patients and how it is related to TCM use. A minority of enrolled PPD women in the present study had a winter delivery in both the TCM user group (21.7%) and the non-TCM group (19.8%). Although the present study did not reflect the real incidence rate of PPD development in different seasons, an earlier study revealed that the risk of PPD development for winter delivery was higher than for deliveries in other seasons [32]. Other studies, however, showed that there is still equivocal evidence in the relationship between the season of delivery and PPD [33–36]. An earlier study also showed that the OR of overall TCM use was highest in winter, in 1997–2013 in Taiwan [37]. However, the season in which delivery was done did not affect TCM use frequency, although patients with PPD that had an autumn delivery seemed potentially higher than those that had a delivery in other seasons (adjusted OR 1.47, 95% CI 0.92–2.36 compared with spring). The reason for this still merits further exploration. In addition, because Taiwan is a narrow subtropical island with limited differences in latitude, such that the climate temperature or day-night variations are not distinct enough during the four seasons, different patterns of CHM of PPD with seasonal variations is still interesting, and further investigation is warranted.

Compared with the residents in Taipei city, residents in central (adjusted OR: 2.05, 95% CI: 1.09–3.86) and southern (adjusted OR: 3.17, 95% CI: 1.58–6.33) Taiwan had a higher TCM use rate. The most historical and famous TCM doctor's education school, China Medical University, was in Taichung in central Taiwan. It offers intact baccalaureate TCM or postbaccalaureate TCM education programs, which delivers most of the qualified TCM doctors in Taiwan [38]. In addition, central Taiwan has the highest density of TCM doctors. The aforementioned university is probably why a particularly higher prevalence of TCM use was observed in central Taiwan. Furthermore, the reason for the higher prevalence of TCM use observed in southern Taiwan might be because southern Taiwan has a greater amount of Chinese herbal medicine pharmacies that provide convenient, traditional, and experienced prescriptions. Culture could be a factor driving residents to establish habits of taking Chinese herbal medicines or visiting TCM clinics.

Beyond expectations, our study shows that combination therapy using TCM and WM for patients with PPD not only caused extra financial burden, it even slightly lowered the burden compared to using simple WM. Even though we could not address direct therapeutic efficacy through the NHIRD files, we assumed the reason that patients use TCM could be to reduce WM clinic visits and western medication use, indirectly proving that TCM is empirically considered a “simple, convenient, cheap, and effective therapy” compared with WM in treating diseases.

However, there are still some limitations in this study. First, the NHIRD data did not contain a symptomatic or biochemical approach for the PPD cohorts. Hence, further studies are required to explain the substantial efficacy of TCM. Second, the database does not contain information on daily activity, dietary habits, and lifestyle, which may also be factors for TCM use and health care costs. Further studies considering the above information are warranted. Third, misclassification bias could be a concern in this study as TCM user classification was done only on the basis of reimbursement by the Taiwanese NHI program. The program only reimburses granular or powder forms of CHM; however, some patients purchase decoction formulations of Chinese medicine directly in TCM pharmacies, which are self-paid and not covered by the NHI program. Therefore, it may be possible that some TCM users were misclassified as non-TCM users and the prevalence of TCM use could be underestimated in the present study. Lastly, we only explored general TCM use, which showed that CHM is the most widely accepted type of TCM. In regard to the PPD and TCM core prescriptions, we are currently analyzing CHM patterns, using animal experiments, and performing clinical studies to discover the regulatory effects and mechanisms.

## 5. Conclusion

The present study is the first report to overview TCM use and prevalence among patients with PPD that underwent treatment with WM in Taiwan. Our study showed that TCM as a complementary therapy combined with WM was accepted by more than half of the patients with PPD, and it potentially could lead to a reduced medical expenditure in treating the disease. The study also discussed the associated sociodemographic and medical factors regarding TCM use of PPD patients. The results of our study may be helpful to clinical practitioners as well as health-policy decision-makers while considering the integration of TCM with WM in patients with PPD.

## Figures and Tables

**Table 1:** Characteristics of patients with postpartum depression according to use of traditional Chinese medicine.

	TCM used						*p* value	Adjusted OR (95% CI)^‡^
	Total (*N* = 653)		No (*N* = 309)		Yes (*N* = 344)			
	*n*	%	*n*	%	*n*	%		
Age mean ± SD (years)^&^	31.2 ± 8.48		31.7 ± 8.91		30.8 ± 8.06		0.22	
Age group							0.10	
20–25	123	18.8	53	17.2	70	20.4		3.98 (1.43, 11.1)^*∗∗∗*^
26–30	211	32.3	102	33.0	109	31.7		3.05 (1.13, 8.21)^*∗∗∗*^
31–35	186	28.5	82	26.5	104	30.2		3.28 (1.26, 8.57)^*∗∗∗*^
36–40	80	12.3	41	13.3	39	11.3		2.30 (0.84, 6.30)
40–45	26	3.98	19	6.15	7	2.03		1.00 (0.32, 3.08)
>45	27	4.13	12	3.88	15	4.36		1.00

Insured amount (NT$/month)							0.49	
<20,000	581	89.0	275	89.0	306	89.0		1.00
20,000–39,999	51	7.81	27	8.74	24	6.98		1.14 (0.60, 2.18)
40,000–59,999	17	2.60	6	1.94	11	3.20		1.83 (0.59, 5.64)
≥60,000	4	0.61	1	0.32	3	0.87		1.40 (0.20, 9.89)

Urbanization^†^							0.45	
Level 1 (highest)	203	31.1	92	29.8	111	32.3		1.00
Level 2	214	32.8	111	35.9	103	29.9		0.81 (0.51, 1.29)
Level 3	101	15.5	47	15.2	54	15.7		0.98 (0.54, 1.78)
Level 4	88	13.5	41	13.3	47	13.7		1.03 (0.53, 1.99)
Level 5 (lowest)	47	7.20	18	5.83	29	8.43		1.86 (0.79, 4.38)

Residential area							0.002	
Northern	85	13.0	54	17.5	31	9.01		1.00
Taipei	256	39.2	126	40.8	130	37.8		1.68 (0.92, 3.07)
Central	113	17.3	46	14.9	67	19.5		2.05 (1.09, 3.86)^*∗∗∗*^
Southern	84	12.9	27	8.74	57	16.6		3.17 (1.58, 6.33)^*∗∗∗*^
Eastern	20	3.06	9	2.91	11	3.20		1.91 (0.61, 5.95)
Kao-Ping	95	14.6	47	15.2	48	14.0		1.71 (0.88, 3.32)

Insured unit							0.21	
Government, school employees	75	11.5	28	9.06	47	13.7		3.07 (1.34, 7.05)^*∗∗∗*^
Private enterprise employees	279	42.8	131	42.4	148	43.2		2.98 (1.46, 6.10)^*∗∗∗*^
Occupational member	159	24.4	75	24.3	84	24.5		3.36 (1.58, 7.14)^*∗∗∗*^
Farmers, fishermen	58	8.90	33	10.7	25	7.29		1.00
Low-income households and veterans, other regional populations	81	12.4	42	13.6	39	11.4		2.26 (1.03, 4.95)∗∗

Season of delivery							0.51	
Spring (March–May)	168	25.7	83	26.9	85	24.7		1.00
Summer (June–August)	180	27.6	87	28.2	93	27.0		1.09 (0.69, 1.74)
Autumn (September–November)	170	26.0	72	23.3	98	28.5		1.47 (0.92, 2.36)
Winter (December–February)	135	20.7	67	21.7	68	19.8		1.03 (0.63, 1.69)

TCM use one year prior to PPD diagnosis							<0.001	
Non-TCM users	369	56.5	230	74.4	139	40.4		1.00
TCM users	284	43.5	79	25.6	205	59.6		4.36 (3.08, 6.19)^*∗∗∗*^

Baseline comorbidity								
Diabetes mellitus^#^	9	1.38	3	0.97	6	1.74	0.51	0.62 (0.18, 2.09)
Hypertension	49	7.50	21	6.80	28	8.14	0.52	0.99 (0.45, 2.18)
Hyperlipidaemia	41	6.28	18	5.83	23	6.69	0.65	1.45 (0.66, 3.21)
Stroke^#^	6	0.92	4	1.29	2	0.58	0.43	0.77 (0.15, 4.13)
Coronary artery disease	48	7.35	23	7.44	25	7.27	0.93	1.23 (0.61, 2.47)
Cirrhosis	107	16.4	47	15.2	60	17.4	0.44	1.10 (0.68, 1.76)
Renal disease^#^	10	1.53	4	1.29	6	1.74	0.76	0.81 (0.24, 2.73)
TCM visits, (mean, SD)					5.58	6.73		

Total medical expenditures (NT$) (mean, SD)	45118	164779	47395	217714	43071	95086	0.74	

Duration between delivery date and diagnosis date, years (mean, SD)	0.44 ± 0.30		0.45 ± 0.30		0.43 ± 0.30		0.71	

Duration between diagnosis date and index date, years (mean, SD)					0.31 ± 0.29			

TCM, traditional Chinese medicine; OR, odds ratio; SD, standard deviation; CI, confidence interval. ^&^*t* test; ^#^Fisher's exact test; ^*∗*^*p* < 0.05, ^*∗∗*^*p* < 0.01, ^*∗∗∗*^*p* < 0.001.^†^The urbanization level was categorized by the population density of the residential area into 5 levels, with level 1 as the most urbanized and level 5 as the least urbanized. ^‡^Adjusted ORs were from the model considering age, urbanization, TCM visit one year ago, insured amount, residential area, insured unit, season, and baseline comorbidity.

**Table 2:** Types of traditional Chinese medicine treatment received in patients with postpartum depression.

Total TCM users	Simple Chinese herbal medicine treatment	Simple acupuncture or manipulative treatment	Combined with both treatment
*n* (%)	*n* (%)	*n* (%)	*n* (%)
344 (100)	246 (71.5)	8 (2.3)	90 (26.2)

TCM, traditional Chinese medicine.

**Table 3:** Top 5 coexisting disease codes among patients after diagnosed postpartum depression for all outpatients visits.

	Non-TCM users			TCM users		
Ranking	Disease (code)	*n*	%	Disease (code)	*n*	%
1	Acute upper respiratory infections of unspecified site (465.9)	15	4.89	Acute upper respiratory infections of unspecified site (465.9)	20	5.83
2	Acute bronchitis (466.0)	10	3.26	Acute nasopharyngitis (common cold) (460)	13	3.79
3	Acute tonsillitis (463)	8	2.61	Acute tonsillitis (463)	12	3.5
4	Other and unspecified noninfectious gastroenteritis and colitis (558.9)	8	2.61	Dental caries (521.0)	11	3.21
5	Acute nasopharyngitis (common cold) (460)	7	2.28	Acute pharyngitis (462)	7	2.04

TCM: traditional Chinese medicine.

## Data Availability

The data used to support the findings of this study are available from the corresponding author upon request.

## Abbreviations

CI: Confidential interval
ICD-9-CM: International classification of diseases, ninth revision
LHID 2000: Longitudinal Health Insurance Database 2000
NHIRD: National Health Insurance Research Database
OR: Odds ratio
PPD: Postpartum depression
TCM: Traditional Chinese medicine
WM: Conventional western medicine.
